# Draft Genome Sequence of *Carnobacterium divergens* V41, a Bacteriocin-Producing Strain

**DOI:** 10.1128/genomeA.01109-16

**Published:** 2016-10-13

**Authors:** Benoît Remenant, Frédéric Borges, Catherine Cailliez-Grimal, Anne-Marie Revol-Junelles, Laurent Marché, Aurélie Lajus, Claudine Médigue, Marie-France Pilet, Hervé Prévost, Monique Zagorec

**Affiliations:** a UMR1014 SECALIM, INRA, Oniris, Nantes, France; b Université de Lorraine, Laboratoire d’Ingénierie des Biomolécules (LIBio), Vandoeuvre-lès-Nancy, France; c Laboratoire d’Analyse Bioinformatique en Génomique et Métabolisme, CNRS-UMR 8030, Évry, France; d Commissariat à l’energie atomique (CEA), Direction de la Recherche Fondamentale (DRF), Institut de Génomique, Genoscope, Évry, France

## Abstract

In this study, we present the draft genome sequence of *Carnobacterium divergens* V41. This strain was previously reported as producing divercin V41, a bacteriocin of interest for food biopreservation. Its genome revealed also the presence of a gene cluster putatively involved in polyketide production, which is unique in lactic acid bacteria.

## GENOME ANNOUNCEMENT

*Carnobacterium* spp. are lactic acid bacteria (LAB) whose taxonomic classification has been modified several times. The *Carnobacterium* genus is currently composed of 11 species that have been isolated mostly from water or sediment and/or cold environments. Among these, *Carnobacterium maltaromaticum* and *Carnobacterium divergens* are also dominant in meat (beef, pork, and poultry), seafood (fish and shrimps), and dairy (raw milk and cheese) food products. These two species can act as food spoilage bacteria or as protective cultures, depending on the strain and on the food product (1, 2). *C. divergens* V41 (CNCM I4028), isolated from fish viscera, produces divercin V41, a class IIa bacteriocin inhibiting *Listeria monocytogenes* (3, 4).

The *C. divergens* V41 genome was sequenced using Illumina HiSeq 2000 technology with the sequencing kit TruSeq SBS version 5 (Illumina, CA). A total of 25,762,368 (50-bp) paired reads were assembled *de novo* with the Velvet software (5) into 32 contigs, ranging from 208 to 775,204 bp, with an *N*_50_ estimated at 386,603 bp. The assembled sequence was 2,743,912 bp, with a G+C content of 35.38%. This genome size is similar to that of other *Carnobacterium* species but smaller than those of *C. maltaromaticum* LMA28 (3.65 Mbp) (6) and ATCC 35586 (3.54 Mbp) (7), although the two species are close (1).

Annotation performed on the MicroScope platform (8) detected 2,589 coding sequences (CDSs) and 38 tRNAs. Upstream from the *dvnVTIRK* gene cluster, which was previously reported as being responsible for divercin V41 production (4), we noticed a CDS similar to PedC, a thiol-disulfide oxidoreductase putatively involved in the posttranslational maturation of bacteriocins (9). No other bacteriocin production genes were identified.

More interestingly, we noticed a 39-kb genomic island not yet reported in LAB, surrounded by transposases suggesting its acquisition through horizontal gene transfer. It encompasses 9 CDSs identified as polyketide synthases/nonribosomal peptide synthases (PKS/NRPS) or PKS/NRPS-like enzymes, a putative regulator of the TetR family, and 3 CDSs encoding putative transporters. This *C. divergens* V41 PKS/NRPS genomic island was similar to that of *Streptococcus mutans* UA59, a pathogen responsible for dental caries (10, 11). Identity scores were 30 to 50% and 57 to 80% with *S. mutans* NRPS/PKS enzymes and transporters, respectively. The molecules produced by such nonribosomal synthesis may have various functions, including antimicrobial or immunomodulatory activities, oxidative stress resistance, or cytotoxicity (12). Some have been reported as pigments without a description of their physiological role. The antiSMASH (antibiotics & Secondary Metabolite Analysis Shell) (13) monomer predicted structure was (pk) + (Mal) + (Leu-Ser) + (Pro-Val-Cys-Gly) + (pk). However, no putative function could be deduced from this predicted structure. Although the production of this unexpected secondary metabolite has not been evidenced, it merits attention especially for the use of *C. divergens* strain V41 in food bioprotection. PCR experiments confirmed that this gene cluster was unique in *C. divergens* V41 among 25 other *C. divergens* strains isolated from different food products.

### Accession number(s).

This whole-genome shotgun project has been deposited in ENA under the accession numbers FLLU01000001 to FLLU01000032. The versions described in this paper are the first versions.
